# Climate, currents and species traits contribute to early stages of marine species redistribution

**DOI:** 10.1038/s42003-022-04273-0

**Published:** 2022-12-03

**Authors:** Jorge García Molinos, Heather L. Hunt, Madeline E. Green, Curtis Champion, Jason R. Hartog, Gretta T. Pecl

**Affiliations:** 1 Arctic Research Center, Hokkaido University, Sapporo, Japan; 2 Department of Biological Sciences, University of New Brunswick, PO Box 5050, Saint John, NB E2L 4L5 Canada; 3 CSIRO Oceans & Atmosphere, Castray Esplanade, Hobart, TAS Australia; 4 Institute for Marine and Antarctic Studies, University of Tasmania, Hobart, TAS Australia; 5 Fisheries Research, NSW Department of Primary Industries, Coffs Harbour, NSW Australia; 6 Southern Cross University, National Marine Science Centre, Coffs Harbour, NSW Australia

**Keywords:** Climate-change ecology, Biooceanography

## Abstract

Anthropogenic climate change is causing a rapid redistribution of life on Earth, particularly in the ocean, with profound implications for humans. Yet warming-driven range shifts are known to be influenced by a variety of factors whose combined effects are still little understood. Here, we use scientist-verified out-of-range observations from a national citizen-science initiative to assess the combined effect of long-term warming, climate extremes (i.e., heatwaves and cold spells), ocean currents, and species traits on early stages of marine range extensions in two warming ‘hotspot’ regions of southern Australia. We find effects of warming to be contingent upon complex interactions with the strength of ocean currents and their mutual directional agreement, as well as species traits. Our study represents the most comprehensive account to date of factors driving early stages of marine species redistributions, providing important evidence for the assessment of the vulnerability of marine species distributions to climate change.

## Abstract (web summary)

Out-of-range observations by citizen scientists and species range data reveal climatic, oceanographic, and ecological drivers of marine species redistributions.

## Introduction

Anthropogenic climate change is driving a rapid, global reorganization of life on Earth with far-reaching consequences for ecosystems and human well-being^1^. Species redistributions towards higher latitudes are particularly evident in the ocean where species more completely occupy their latitudinal range within their thermal limits^2^, have greater physiological vulnerability to environmental warming^3^ and experience less barriers to dispersal^4^. However, climate expectations based solely on long-term, gradual warming fall short of explaining the wide variability observed in range shift responses^5^. Multiple factors can interact with climate warming to drive range shifts, including climate extremes^6^, oceanographic conditions^7^, species traits^8^ and their interactions^9^, habitat constraints^10,11^, and human activities^12^. However, evidence remains fragmented and more comprehensive, multifactor studies are needed to improve our understanding of the drivers of species range shifts under climate change^13^.

**Fig. 1:**
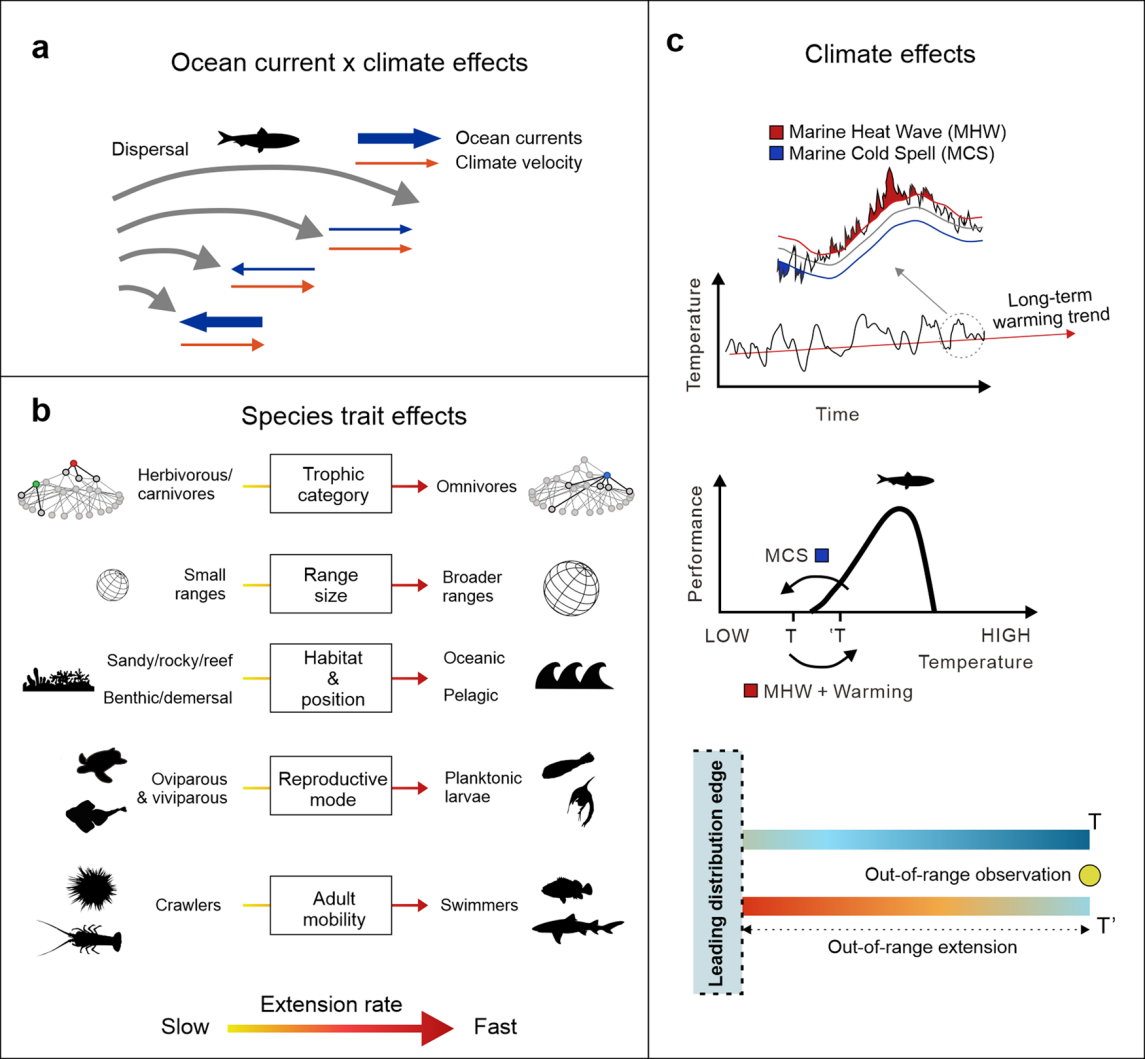
Schematic of a priori expectations for the role of physical and biological predictors in driving extension distances associated with out-of-range observations. **a** Through their effect on dispersal, ocean currents may facilitate or hinder this process depending on their transport capacity (represented by the thickness of the blue arrow) and their directional agreement with the displacement of thermal environments under warming. **b** Biological identity is also expected to influence responses to warming based on traits related to the dispersal potential of adults and larvae, dependency on specific habitats, trophic category and historical range size (see text for details). **c** Dispersal of individuals beyond the leading, cooler edge of a species distribution are expected to respond to extensions of its thermal niche under the combined effects of long-term, gradual warming and transient, episodic events (marine heat waves, MHW) as areas outside of the distribution initially too cold for the species (T) become thermally suitable (T’). Marine cold spells (MCS) may hinder or halt extensions by locally setting ambient temperatures below the species lower tolerance limits.

Here, we provide the most complete assessment to date of the factors driving early stages of marine species redistributions. Our assessment looks at the combined effect of long-term warming, short-term climatic extremes (i.e., heatwaves and cold spells), the strength of ocean currents and their directional agreement with climate warming, and species traits in predicting maximum annual out-of-range distances for an Australian dataset of marine fishes, reptiles, and invertebrates. Out-of-range observations represent the arrival stage of range shifts^14^, and have been shown to be linked to climatic features for marine species^15^ and birds^16^. We hypothesized that out-of-range observations of species occur further from the poleward range edge of their historical distribution where the displacement of thermal environments associated with long-term climate warming is faster^8,17–20^, directionally aligned with ocean currents^7,19^, and where ocean transport is strongest^20^ (Fig. 1a). Species responses to climate warming are also contingent on differences in their life history and ecological traits. For example, faster range shifts are expected for species with traits related to greater dispersal potential (e.g., swimming ability) and ecological versatility (e.g., greater latitudinal range size and omnivory^8^; Fig. 1b). The initial arrival stage of range extensions (the stage examined in this study) is expected to depend on dispersal-related traits while the establishment stage of range shifts may be more linked to the annual temporal persistence of suitable environmental conditions^21^ and the ability of range-shifting species to exploit local food resources^22^. Finally, over and above the effect of gradual warming, temperature extremes can also cause episodic but drastic displacements of thermal habitats that can lead to large, relatively quick shifts in the distribution of marine species^6,23,24^. Marine heatwaves (MHWs) are thus expected to facilitate species range extensions at rates greater than that induced by gradual warming trends^25^. Similarly, marine cold spells (MCSs), whose effects are much less explored than those of MHWs, can create local thermal conditions that are below species lower thermal tolerances, effectively limiting or even reversing their initial dispersal and subsequent stages of range extensions^26^ (Fig. 1c).

**Fig. 2:**
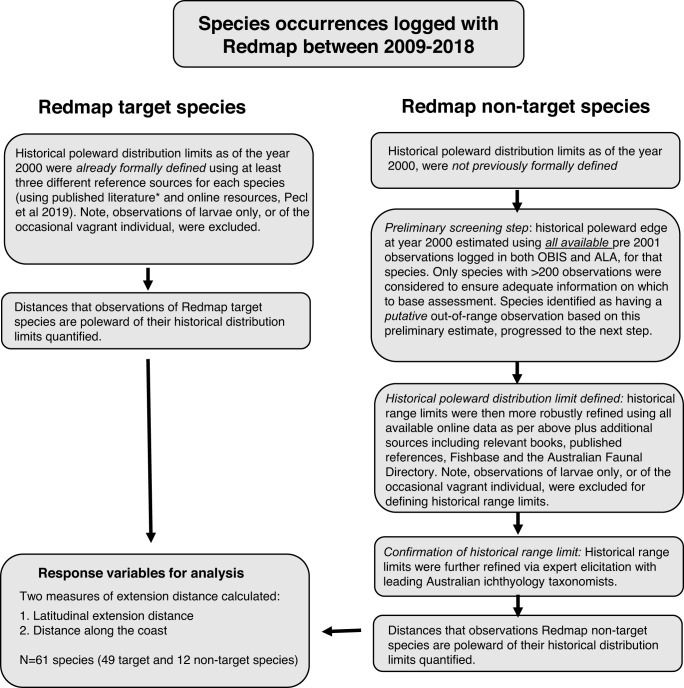
Flow diagram of the steps taken in the estimation of historical distribution limits of species. Citizen scientists can can submit observations to the Redmap program of species from region-specific lists of ‘target’ species that are unusual to particular areas (i.e., outside previously and systematically determined expected historical distribution limits) as well as observations of any species they consider unusual for an area (non-target species).

We tested these hypotheses on a data set of annual maximum out-of-range distances (*n* = 127) calculated both as latitudinal distance (i.e., direct latitudinal extension distance) and distance along the coastline (i.e., coastal extension distance) relative to the historical range boundaries of 61 species of marine fishes, reptiles, and invertebrates. Our objective was to utilize multiple sources of historical distributional information (Fig. 2) as a baseline for estimating extension distances for out-of-range citizen science observations. Our dataset encompasses observations recorded along the south-eastern and south-western coasts of Australia between 2009 and 2018, which are two of the fastest warming regions in the southern hemisphere^27^. Due to the general arrangement of the coastlines, the direction of warming, and the presence of poleward-flowing currents, the primary focus of the study was on latitudinal shifts. Data were sourced from the Range Extension Database and Mapping citizen science program Redmap Australia (hereafter ‘Redmap’; see Supplementary Data 1 and www.redmap.org.au^28^). Our suite of physical and biological predictors (Supplementary Data 1) comprised (1) the velocity of climate change (*sensu*^17^) to capture the effect of background long-term, gradual warming, (2) the effect of acute, episodic warming or cooling events reflected by the thermal displacements associated with MHWs^29^ and the cumulative intensity of local MCS^26^, (3) the strength (kinetic energy) of ocean surface currents and their directional agreement with warming^7^, to capture the effect of ocean currents on the capacity of species to track the shifting thermal environments, and (4) a suite of biological traits thought to influence species range shift responses (see Methods and Supplementary Data 2).

## Results

### Range extension distances

**Fig. 3:**
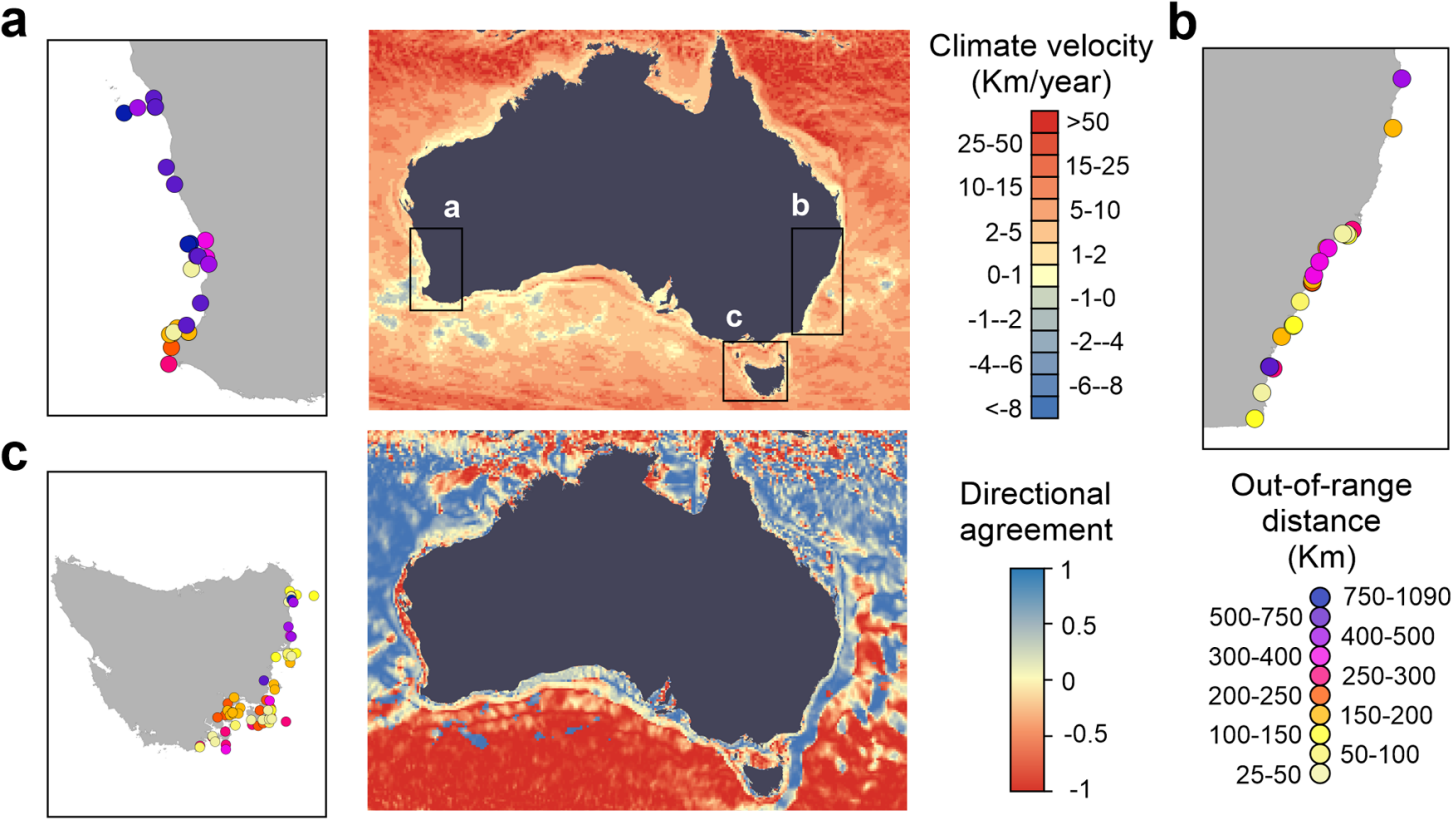
The velocity of climate change, its directional agreement with ocean surface currents, and the locations of out-of-range observations. The velocity of climate change and its directional agreement with average mean annual surface currents (1994–2018). Inset panels show the out-of-range observations from (**a**) western Australia, (**b**) eastern Australia and (**c**) Tasmania (*n* = 127) colored by their corresponding maximum annual coastal extension distances relative to the historical southern distribution limit of each species. Note that multiple observations for the same species over consecutive years are possible along the same coastline (see Methods for details).

Maximum extension distances varied considerably among observations (latitudinal extension distance: 246.4 ± 194.0 km; coastal extension distance: 343.2 ± 315.3; mean ±1 SD) even at relatively small spatial scales (Fig. 3a–c), with some individuals detected up to 1099 km poleward of their historical range limit (or 1802 km if measured along the coast; see Supplementary Data 1). Geographically, the west coast of Australia registered the highest mean extension distances (Fig. 3a), while the east coast of Tasmania accumulated the highest number of observations (Fig. 3c). As expected, given the presence-only nature of opportunistic observations (i.e., absence of sampling effort information), no clear temporal trend in range extension observations was apparent during the study period (2009–2018). Nonetheless, out-of-range observations were detected for the largest number of species (*n* = 28) in 2016. Climate velocity calculated over the last 25 years (1994–2018) was positive throughout most of the study domain, indicative of widespread sea surface warming (Fig. 3). Poleward flowing currents on the east (i.e., the East Australian Current) and west (i.e., the Leeuwin Current) coasts of mainland Australia resulted in positive directional agreement between climate velocity and ocean currents in most coastal areas, although directional agreement was negative in nearshore areas along the west coast of mainland Australia and some parts of Tasmania (Fig. 3) likely due to localized oceanographic processes.

Climatic extreme metrics had overall high values across the study regions with no obvious spatial pattern (Supplementary Fig. 1). Median thermal displacements caused by MHWs at the southern distribution edge of each species over the 10 years previous to each maximum annual out-of-range observation had an average value of 121.6 ± 103.5 km (mean ± 1 SD), ranging from 0 (no MHW events) to 509.5 km (Supplementary Fig. 1; Supplementary Data 1). Cumulative intensity of MCSs occurring over the same 10-year period throughout the estimated extended range of each species also varied widely (0–856.6 °C day) with an average of 136.5 ± 160.0 °C day.

### Relationships between extension distances and oceanographic variables

**Fig. 4:**
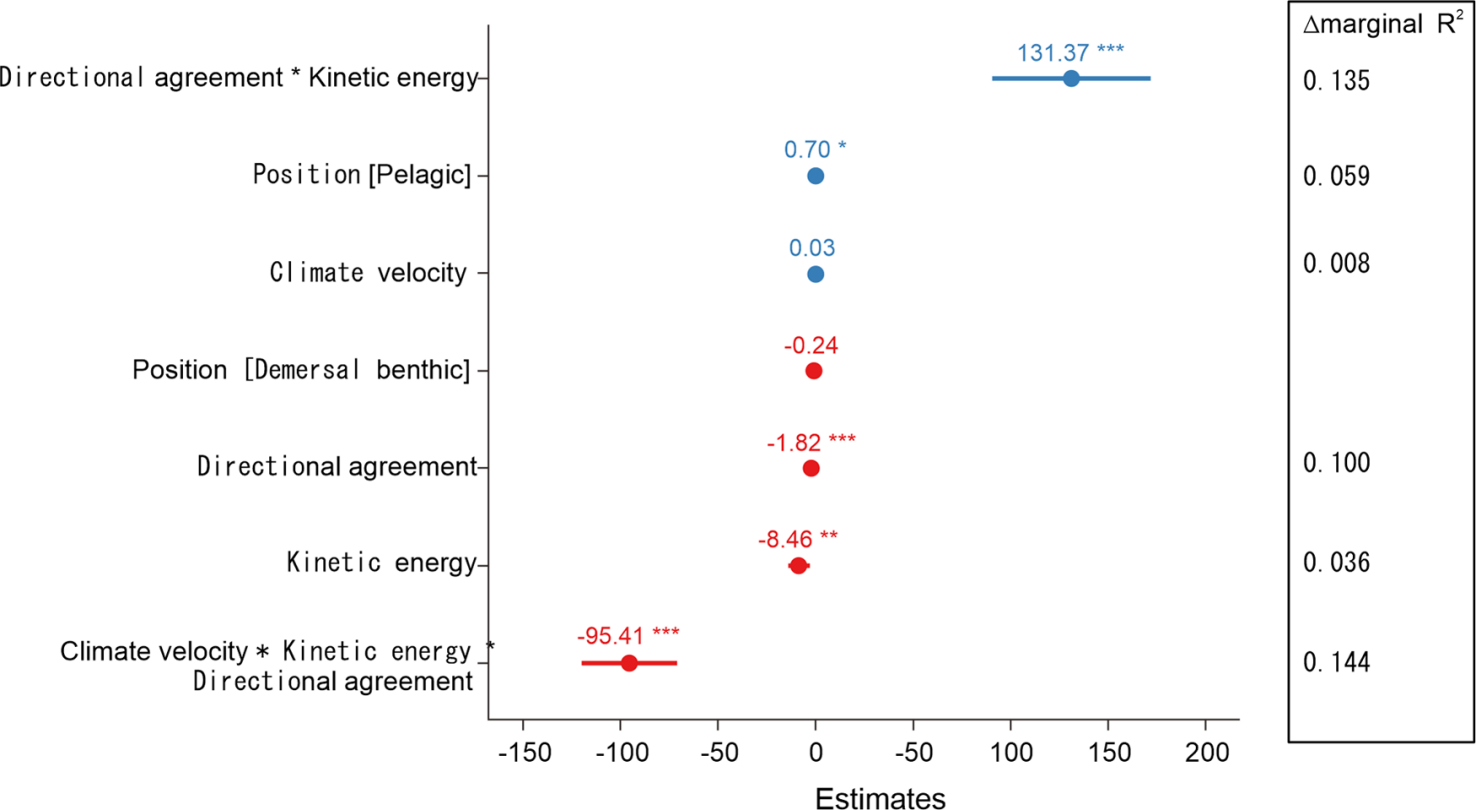
Regression coefficients and relative change in marginal *R*^2^ for the terms retained in the final model for prediction of maximum annual extension distances along the coast. Estimates of regression coefficients with corresponding 95% confidence intervals for each main and interaction term retained in the final log-linked generalized linear mixed model (GLMM). Regression coefficient estimates are on a log_10_ scale. The inset panel shows relative change in marginal (fixed effect) *R*^2^ resulting from the removal of that particular term from the final model as an indication of its relative contribution towards the total observed variance in response explained by the selected model. *n* = 127 maximum annual extension distances.

**Fig. 5:**
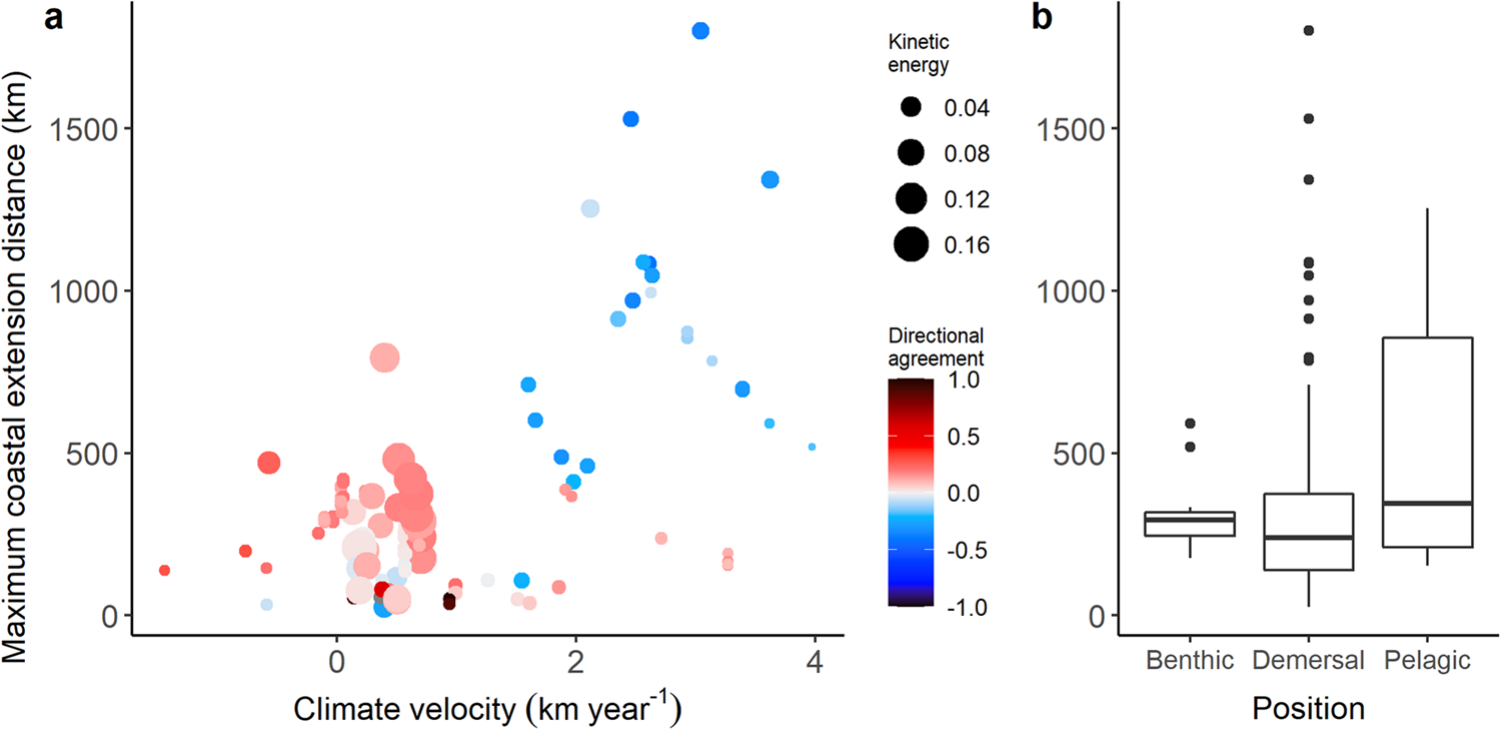
Relationships between observed maximum annual extension distances along the coast and the predictor variables retained in the final model. **a** Scatterplot of extension distance and climate velocity with colors and size of the points showing the directional agreement between climate velocity, ocean currents and current strength, respectively. **b** Box plots (center line, median; box limits, 75th and 25th percentiles; whiskers, 1.5× interquartile range; points, outliers) of extension distance grouped by position in the water column of the species. *n* = 127 maximum annual extension distances.

Model selection on the full model (see Methods and Supplementary Table 1) yielded final models (Fig. 4; Supplementary Table 2; Supplementary Fig. 2) with 48% and 57% of the total variance in maximum extension distances accounted for by the fixed effect component of the model (i.e., the predictors) for latitudinal and coastal extension distances, respectively. For the model based on coastal extension distance (Fig. 4), the three-way interaction between climate velocity, current kinetic energy, and directional agreement between currents and thermal gradients had the highest relative importance (14% reduction in marginal R^2^ resulting from its removal from the model), followed by several other physical predictors: the interaction between directional agreement and kinetic energy (−13 %), and the main effects of directional agreement (−10%) and kinetic energy (−3%). Climate velocity was also retained in the model because of the significant three-way interaction, but the main effect of climate velocity was not statistically significant (Fig. 4). Similar results were attained for the model based on latitudinal extension distances, although relative importance was more evenly distributed among the physical variables and their ranking was slightly different from the model based on coastal extension distances (Supplementary Fig. 2). Overall, for both latitudinal and coastal extension distances, there was a positive relationship between extension distances and climate velocity, although the significant three-way interaction indicated that relationships between extension distances and climate velocity, directional agreement, and kinetic energy of currents were complex. Large range extensions occurred in locations where high climate velocity displaced thermal niches quickly. For example, observations linked with mean climate velocities >2 km y^−1^ were associated with mean coastal and latitudinal extension distances of 752.9 ± 464.7 km and 473.52 ± 269.4 km, respectively. This relationship occurred even in situations where current speeds were low and did not match the direction of climate velocity (i.e., negative directional agreement; Fig. 5a; Supplementary Fig. 3a). For observations associated with high values of kinetic energy (>0.05 m^2^ s^−2^), extension distances were positively related to directional agreement (Fig. 5a; Supplementary Fig. 3a).

### Effect of species traits on species redistributions

Position in the water column was a significant predictor of coastal extension distances (6% reduction in marginal R^2^; Fig. 4), with greater distances associated with pelagic than demersal or benthic species (Fig. 5b). Trophic category was a significant predictor of latitudinal extension distances (Supplementary Fig. 2), with carnivores and omnivores having greater extension distances than herbivores (Supplementary Fig. 3b). Other species traits (adult mobility, range size, reproductive mode and habitat) did not have significant effects on extension distances for either of the two dependent variables (Supplementary Table 1).

## Discussion

Our study offers the most complete understanding to date of the physical and biological variables that contribute to early stages of species range extensions in the ocean. Previous research has demonstrated the importance of accounting for factors other than temperature when predicting distribution shifts in response to climate warming^7,8,10,30,31^. For example, an assessment of range extensions for 89 fish and invertebrate species of south-eastern Australia^8^ found that incorporating species traits in a climate-expectation model informed solely by climate velocity increased the proportion of total explained variance from 23.1% to 57.8%. Similarly, a recent global meta-analysis^7^ of marine distribution shifts found that a model informed by climate velocity and directional agreement of currents increased the explained variance by 25% relative to the climate-expectation model (i.e., from 41% to 66%). Our final model explained a comparable amount of variability in species range extensions (57% and 48% for coastal and latitudinal extension distances, respectively). However, unlike previous studies, we found complex interactive effects between climate velocity, directional agreement, and current speed on extension distances of marine species along both the south-eastern and south-western coasts of Australia.

While the largest observed coastal and latitudinal extension distances were consistently associated with large climate velocities, even in the presence of mild to medium directional mismatch with currents, relatively large extension distances were still associated with medium to low climate velocities under positive directional agreement, particularly when the transport capacity of ocean currents was high (Fig. 5a). These results suggest that climate warming exerts a strong effect on shift responses in these marine species, overriding the opposing effect of directionally mismatched currents.

Many of the analyzed species are fishes with active adult dispersal capacity capable of countering the effect of opposing currents, particularly when they are relatively weak as in our case (Fig. 5a). On the other hand, where strong currents align with the direction of climate warming, species may achieve large extension distances, in some cases “going ahead of the climate” and overcompensating for the warming effect. This climate overcompensation effect mediated by currents has also been reported in a previous global meta-analysis^7^. Our results indicate that both current speed and alignment of ocean currents with thermal gradients influence early stages of warming-driven range extensions, which has implications for rates and geographic patterns of biodiversity redistribution in the ocean. More evidence covering a wider gradient of combinations between these three parameters and a more diverse pool of taxa is needed to better understand the interactive effects between climate velocity, current speed, and directional alignment.

Including species traits slightly increased the amount of variation explained by our models (Fig. 4, Supplementary Fig. 2). Position in the water column and trophic category were retained in the final models for coastal and latitudinal extension distances, respectively, although their effects were similar for both extension distance metrics. Greater distribution shifts for pelagic than benthic species have been observed for fish in the U.S. Northeast Large Marine Ecosystem^11^, with species whose distributions are primarily associated with oceanographic variables shifting larger distances relative to those whose distributions are determined by substrate or depth. The greater latitudinal extension distances of predators and omnivores than herbivores in our study contrasts with a previous study of marine species in south-eastern Australia^8^ that found range extension rates were greatest for omnivores and lowest for predators. However, the importance of omnivory in Sunday et al.’s study^8^ was driven mainly by a small number of species. Predators may have faster range extensions than lower trophic levels as they are often generalists that are less sensitive to prey type^8^ and they are generally more mobile than their prey^32^. The lack of a significant relationship of coastal and latitudinal extension distances to range size was unexpected. Previous studies^8^ have found range size to be positively related to range extension rates, consistent with hypotheses that broad ecological tolerances are important for range extensions^8^, or that broader latitudinal ranges may generally have greater local abundance and therefore exert greater propagule production, leading to faster range extensions^33^. Nonetheless, previous studies have found inconsistent relationships between species’ traits and range shifts^8,20,34^. The small amount of variability explained by some of the species traits in our models is likely due to the majority of the species falling into a single category for those traits (life history [planktonic larvae], mobility [high adult mobility], habitat [reef-associated]).

Neither the displacement of local thermal conditions at the distribution edge of the species caused by marine heatwaves nor the cold spells that occurred throughout the expanded range during the 10 years previous to the documented range extension were retained in the final models. This outcome was unexpected as climate extremes are undoubtedly important factors driving the redistribution of marine species in coastal environments^6,23,26^, and most of our study region was subject to some high- magnitude MHWs during the period spanning our observations, such as the devastating MHWs that occurred in Western Australia in 2010–2011^35^ and the Tasman Sea in 2015–2016^36^. It is possible that the metrics we used to capture the effect associated with climate extremes or the temporal and spatial extent on which they were calculated were mismatched with our response variables. Nonetheless, repetition of the analysis using alternative metrics (e.g., cumulative and mean intensity, frequency and duration of extreme events within the coastal buffer along the distance shifted) did not change the results with regard to the effect of climatic extremes on early-stage range extensions. Further studies are needed to develop and test suitable metrics to account for the effects of climate extremes on the prediction of early range extensions.

Methodological differences among studies have also been shown to account for a large proportion of variation in climate-driven range shifts in marine species^30^. Given the nature of the presence-only opportunistic data used in this study, spatial bias in the distribution of human observers may have contributed to unexplained variation in extension distances. Greater variability in range extensions is also likely to occur at the arrival stage of extensions, as examined in this study, compared to later stages where populations become increasingly established and range expansions consolidate. In contrast to other studies, we analyzed both latitudinal and coastal range extension distances and found that our predictors explained more variance for the latter response variable. Despite these two metrics being highly correlated (*r* = 0.97) in our study given the predominant north-south coastal orientation and our exclusion of predominantly longitudinal shifts (see Methods), using coastal distances as response variable still resulted in an obvious improvement of the model (+9% in marginal R^2^). This result suggests that considering coastal distances for the analysis of range shifts in shallow water species may be particularly informative in situations where they differ greatly from latitudinal distances such as with complex coastal configurations or east-west orientations.

Structured survey data is not available globally at the spatial and temporal scales necessary to track climate-driven redistributions of marine species. Given the pervasive but highly variable nature of range shifts, and the high number of people observing and documenting wildlife daily, the potential for citizen scientists to play an effective role in the early detection of range shifting species, specifically range extensions, is substantial^37^. Here we have demonstrated that out-of-range species observations collected by the public can be used to explore the factors that drive geographic and species-specific variation in early stages of marine range extensions. Citizen science approaches such as Redmap can not only provide quality cost-effective data but can also improve climate change communication and engagement with the public^38^.

By identifying the contributions of physical variables and species traits to the early stages of climate-driven redistributions, this study refines our understanding of regions of the global ocean, and the species occupying these regions, that are likely to be experiencing rapid species redistributions. Global ocean warming hotspots are known to be locations where the early indications of climate-driven range shifts in marine taxa are most apparent^39^. However, we show there is a complex interaction between climate velocity, kinetic energy of currents, and directional agreement between climate velocity and prevailing currents, indicating that multiple physical variables contribute to the early stages of range extension. Given that these physical variables can be derived from remotely-sensed data sources, our results may facilitate a global assessment of ocean regions most likely to be associated with rapid early stages of species redistributions, integrating variables other than changes in sea surface temperature alone^27^. A limited number of species traits (position in the water column or trophic level) also influenced extension distances, although the small amount of variation explained by species traits in this and most other studies^34^ suggests that future research should consider whether alternative traits could be better predictors of range extensions. Our findings may be used to refine predictions of species undergoing early stages of climate-driven range extensions in regions where the physical characteristics of the marine environment are facilitating species redistributions. By providing the most complete understanding of the factors implicated in the early stages of marine species redistributions to date, we improve capacity for correlative and trait-based assessments of the vulnerability of marine species distributions to climate change.

## Methods

### Redmap data and extension distances

Out-of-range observations of marine species in Australia were obtained from Redmap (www.redmap.org.au). Redmap is a citizen science project that requests submission of photographs and associated data for ‘unusual’ observations of marine species (i.e., out-of-range observations) in Australia to a website or smartphone application. The goals of Redmap are (i) monitoring of early indications of potential range shifts of marine species and (ii) engagement and communication with the community in relation to marine climate change using their own data^28^. Redmap began as a pilot project in Tasmania in 2009 and expanded nationally in late 2012. Two types of observations can be logged in Redmap. Contributors to Redmap can submit observations of species from region-specific lists of ‘target’ or ‘listed’ species that are unusual to particular areas (i.e., outside previously and systematically determined expected historical distribution limits). Contributors can also submit photographs of any species they consider unusual for an area (referred to as ‘non-target’ or ‘other’ species). The Redmap program is able to efficiently collect and process species observation data at a national scale due to semi-automated managed crowd-sourcing of a network of 80 taxonomic experts from 26 institutions across the country^28^. Photographs of each observation are sent to one of the experts to verify species identification and provide feedback to the observer. Presently, 220 species are listed as target species in Redmap. Lists of target species were developed for each region by biogeography experts in collaboration with marine users (scientists, resource managers, fishers, and divers). To be considered as a target species, species needed to have a well-defined historical distribution (based on peer-reviewed literature, species observation databases and museum-based checks on species distributions), and to be easily identifiable from an image only (i.e., no easily confused with similar species) and observable (i.e., not cryptic or camouflaged). Historical species distributions of established populations of target species were formally defined using at least three different reference sources for each species using published literature and online resources^28,40^. These historical distributions were defined as of the year ~2000, to allow a ten-year time period between the historical baseline and the Redmap observation dataset, and excluded observations of larvae only, or of the occasional vagrant individual. The 1468 sightings of listed target species submitted to Redmap from 2009–2018 were examined by experts for verification of identification and, if confirmed, compared to their previously defined historical distribution range (Fig. 2). Because the focus of this study was poleward range extensions and for comparability with the method used to estimate the historical distribution limits of non-target species (see next paragraph), only observations that were south (poleward) of the southern limit of a given species within the region of observation were retained for analysis. Regional southern range limits were determined in ArcGIS Pro from the polygon of the historical species distribution as poleward range limits of many of the species differed between the south-eastern and south-western coasts of Australia^28^.

Observations of non-target species in Redmap provide additional out of range observations of marine species in Australia. A total of 1101 sightings of non-target species were submitted to Redmap from 2009–2018, comprising 699 species. Identifications of species were provided by the same taxonomic experts as for the listed species. Only observations identified to the species level were retained for analysis in this study. Historical ranges of these non-target species had not been previously defined by Redmap. Data sourced from online repositories, including the Ocean Biodiversity Information System (OBIS; http://www.obis.org.au) and Atlas of Living Australia (ALA; www.ala.org.au) were used in a preliminary screening step to identify species with putative out-of-range observations (Fig. 2). Only occurrences logged before 2001 were used as a preliminary estimate of species historic range limits to align with the time frame for which historical distribution limits were estimated for target species. Only species associated with ≥200 observations in OBIS/ALA in this time frame were included. Occurrences from OBIS were accessed using the R Package *iobis/robis*^41^ while data from ALA were downloaded directly from the website. In both cases, polygons defining the south-eastern and south-western coasts of Australia were used to limit the extraction of observations within our study region (Supplementary Fig. 4). The maximum latitude of each species was determined separately for the east and west coasts of Australia. Species that were identified as having a putative out-of-range observation (see next section) based on the preliminary estimate of the historical distribution limit were further investigated (via Fishbase^42^ and the Australian Faunal Directory^43^) and evaluated by scientists with expertise in taxonomic, biodiversity and fisheries fields (Fig. 2). Using an approach similar to listed species^28^, the historical distributions of these non-target species were updated using multiple references sources for each species drawn from published literature and online resources (Supplementary Data 2). The use of multiple evidence sources for assessing historical distribution limits and range shifts is considered best research practice in the absence of systematic monitoring of range dynamics^14^. Similar methodological processes have been used for defining historical distribution limits in a number of studies e.g., refs. ^8,14,44^. These expert-defined historical distributions were used to determine whether the putative out-of-range observations of non-target species were range extensions.

Contributors to Redmap can select a spatial scale to record their observation in order to respect their need for confidentiality and protect locations of threatened species. Observations recorded at scales of 10 m–10 km were used in our analyses while those recorded at a coarser scale (an option provided during 2009–2012 in Tasmania) were excluded. Observations from northern Australia (i.e., north of 28°S) were excluded due to the insufficient number of citizen science observations in northern regions. Queensland has only recently been added to Redmap and the northern portion of Western Australia is very sparsely populated and has very low levels of fishing and diving along its coastline^28^.

We excluded multiple extension observations per year of a given species as they could be re-sightings of the same individual or result from the same settlement event. The maximum extension distance of each species in a given year formed our response variable in the analyses. Maximum out-of-range extension distances were calculated in two different ways (Supplementary Fig. 5). First, latitudinal distances were calculated as the difference in latitude between each observation and the historical southern latitude for that species in a given region, and converted from degrees latitude to km using the ‘pointDistance’ function in the *raster* package^45^ in R. Only sightings >20 km from the maximum southern latitude were considered as range extensions in order be conservative due to the possibility of lack of detection of rare individuals when determining the maximum southern latitude. Second, distances were also calculated as distances along the coast between the historical southern distribution limit of the species and the corresponding maximum annual out-of-distance observation. Given all historical limits for the species considered here are located on the east and west coasts of Australia, distribution limits were simply calculated as the points where the estimated historical southern latitude of the species intersected the coast for the corresponding region. Distances were then calculated as least-cost-path distances using a 5-km resolution transition matrix with null transition cost for the 10 km wide strip of cells along the coast and a very high (10^6^) cost for other marine cells. This assured that path routing between the origin (historical range limit) and destination (out-of-range observation) followed the coast whenever possible yet allowed transitions through open water if needed to reach the destination point (i.e., between mainland Australia and Tasmania). Least-cost-path distances were calculated with the ‘shortestPath’ function of the ‘gdistance’ R package^46^. Nine of 136 maximum annual out-or-range observations were excluded from analysis as they had a predominantly longitudinal component (i.e., longitudinal out-of-range extension » latitudinal extension; *n* = 7) or because the path followed between mainland Australia and Tasmania was unclear (*n* = 2), making determination of a path routing uncertain. Variance explained by the models (see Statistical Analyses) increased when these observations were removed.

### Species traits

Six species traits (trophic category, reproductive mode, adult mobility, range size, water column position and habitat) were selected to test our hypothesis that species with traits related to greater dispersal potential and ecological versatility have out of range observations further from their historical poleward range limit. Information was obtained from a number of sources (Supplementary Data 2). Range size was estimated from the maximum latitudinal extent of observations of a species from OBIS prior to 2001. Trophic category was defined based on diet descriptions as herbivores, omnivores or carnivores. Life history was classified in terms of reproductive mode, which was categorized as highly dispersive with planktonic phase (planktonic), oviparous with no planktonic phase (non-planktonic), or viviparous (live bearing). Adult mobility was defined as high mobility (swimming) or low mobility (crawling). All fish were swimmers and only three species had low adult mobility: the longspine sea urchin (*Centrostephanus rodgersii*), eastern king prawn (*Melicertus plebejus*) and eastern rock lobster *(Sagmariasus verreauxi*). Habitat preference was determined based on adult preferences of each species and categorized as either inshore/sandy, rocky, reef or oceanic. Water column position was described as either exclusively bottom dwelling (benthic), spending time near the bottom (demersal/benthic) or swimming freely in the water column (pelagic).

### Climate velocity, kinetic energy, directional agreement, and climate extremes

Climate velocities^17^ were calculated over the 25-year (1994–2018) annual minimum mean monthly SST series prior to bilinear interpolation to a common 0.2° grid cell resolution to match the coarser resolution of the Integrated Marine Observing System (IMOS) Ocean Current Gridded (0.2° × 0.2°) data set (https://portal.aodn.org.au/ accessed 20 August 2018; Supplementary Table 3). Eddy kinetic energy was derived from altimetry processed by CSIRO Oceans and Atmosphere with 0.2° spatial resolution and annual (1993–2018) averages were calculated. Surface current direction, used to calculate the directional agreement between surface currents and climate velocity (see below), was calculated from the annual averages (1993–2018) of mean daily surface geostrophic velocities from the IMOS data set. The directional agreement between ocean flow and climate velocity was then estimated as the cosine of the angle difference^7^. This index ranges from −1 to 1 for opposite and matching directions, respectively. Current angles were calculated from the annual geostrophic velocities averaged over the 10-year period prior to each observation record. While a 25-year period was selected for climate velocity to capture the long-term gradual warming trend, we calculated current direction and the climate extreme metrics (see below) over a 10-year period to capture the direct influence of ocean flow on dispersion and range extensions of marine species over the shorter time-scales associated with range shifts.

We used two different metrics to capture the expected dual effect of temperature extremes in driving out-of-range expansions at leading edges of a species distribution (Fig. 1) related to the rate of displacement of their thermal habitat produced by (i) marine heat waves (MHWs) and (ii) marine cold spells (MCSs). The thermal displacement associated with MHWs was defined by the spatial shifts of surface temperature contours driven by MHW events^29^. Briefly, their estimation involved the calculation of (i) the 1982–2011 mean monthly climatology from the OISSTv2 daily data, (ii) the linearly detrended monthly temperature anomalies for the period of interest, (iii) the identification of MHWs for each cell over that period, defined as months where SST anomaly exceeds a seasonally adjusted 90th percentile threshold, and (iv) the calculation of the thermal displacement associated with each MHW event by quantifying the distance to the geographically closest cell that has a temperature equal to or less than the baseline temperature (i.e., the observed SST minus the SST anomaly) for the corresponding month from the focal cell experiencing the MHW. Though there are other justifications^29^, in our case the choice of monthly data for the definition of MHWs and calculation of the associated thermal displacement relate primarily to our intention of using this metric as a predictor for maximum annual out-of-range observations of marine species, to which MHWs defined on shorter durations (i.e., daily) is less meaningful. Similarly, the use of linearly detrended, monthly SST anomalies is particularly relevant to our analysis to distinguish the effect of discrete, transient MHWs on range shifts from that of long-term, gradual warming captured by the climate velocity metric. Second, we use the cumulative intensity of MCSs, calculated as the cumulative sum of the average intensity by the duration of each identified event (°C days), to assess the potential local cumulative effect of extreme low temperatures to limit extensions beyond historical species distributions. MCSs were calculated for each cell using the time series of SST daily anomalies for the period of interest relative to the same baseline climatology (1982–2011) as periods of at least five consecutive days with anomalies below a seasonally varying 10th percentile threshold^47^.

For each extension observation, all parameters other than climate velocities were calculated for the 10-year period prior to the year observations were reported. Single estimates for the different parameters (climate velocity, directional agreement, kinetic energy and cumulative intensity of cold spells) were then calculated for each observation by averaging over all cell values within a 25-km buffer following the contour of the coast and spanning between the southern distribution limit and the out-of-range observation (Fig. 1). We selected a buffer width of 25 km to adequately capture the conditions of the relatively shallow coastal habitats occupied by our study species. We used thermal displacement as a proxy for the shift response of a species to the changes in local thermal conditions triggered at its range edge by a heat wave and calculated average thermal displacements associated to MHWs from the median displacements from all months with an active MHW at each cell corresponding to the leading distribution edge of the species (Fig. 1). We used median instead of mean thermal displacement to account for the right skewness associated with the distribution of extremes^29^. Climate velocity and marine cold spells were calculated in R using the ‘VoCC'^48^ and ‘heatwaveR'^49^ packages, respectively. Thermal displacement associated with marine heatwaves were estimated in MATLAB using a modified version of the scripts provided by Jacox et al.^29^.

### Statistics and reproducibility

Analyses were run on 127 maximum yearly out-of-range observations of 61 species (49 target species and 12 non-target species). We used gamma-distributed generalized linear mixed effects models with a log-link to test for the combined effect of physical variables and species traits in predicting observed extension distances. The maximum extension distance of each species in a given year formed our response variable in the analyses. Analyses were carried out separately for latitudinal extension distances and for extension distances along the coast. Our full model for both dependent variables (latitudinal and coastal extension distances) included climate velocity, kinetic energy of currents, directional agreement between climate velocity and currents, MHW thermal displacement, cumulative intensity of MCS, and all species traits as fixed effects. The model included the interaction between climate velocity and local directional agreement between currents and climate velocity, the interaction between directional agreement and kinetic energy of currents, and the three-way interaction between climate velocity, local directional agreement between currents and climate velocity, and kinetic energy of currents. We also included an interaction between climate velocity and thermal displacement to account for the combined effect of trend- and event-focused effects on dynamic thermal environments. Because populations at the leading, cold edge of distributions are expected to be at or close to the species’ lower physiological thermal tolerances (Fig. 1), we used minimum annual mean monthly temperatures for calculating climate velocities and related directional agreement. Models fitted with metrics calculated using mean annual monthly temperatures explained less variation in extension distances. Model selection was performed by removing one variable at a time from the full model using the criteria of largest *p*-value and lowest reduction of marginal R^2^ (Supplementary Table 1) in the resultant sub-model until all parameters in the final reduced model were significant. When there was a significant interaction, main effects were also retained in the model even if they were not all individually significant. Species identity was added as a random effect on the intercept to account for non-random variation in extension distances among species. Visual examination of the full model residuals did not reveal patterns of spatial autocorrelation; therefore, inclusion of a spatial covariance structure was not deemed necessary. Models were fitted with the ‘glmmPQL’ function of the package ‘MASS'^50^ and model performance was assessed with the ‘tab_model’ function of the package ‘sjPlot'^51^.

### Reporting summary

Further information on research design is available in the Nature Portfolio Reporting Summary linked to this article.

## Supplementary information

Supplementary Material

Description of Additional Supplementary Files

Supplementary Data 1

Supplementary Data 2

Reporting Summary

## Data Availability

All source data for the analyses are included in the Supplementary Data files for this article. All other data are available from the corresponding author on reasonable request.

## Code availability

Statistical analyses were carried out using R version 4.0.2. All R packages and functions used for analyses are listed in the Methods of the article. Thermal displacements associated with marine heatwaves were estimated in MATLAB using a modified version of the scripts provided by Jacox et al.^29^. The modified code is available on request.
