# Supplementary material for: Climate, currents and species traits contribute to early stages of marine species redistribution
Source: Commun Biol. 2022 Dec 3;5:1329. doi: 10.1038/s42003-022-04273-0 (PMC9719494; doi:10.1038/s42003-022-04273-0)
Supplement: Supplementary file 1 — Supplementary Material [file 42003_2022_4273_MOESM1_ESM.pdf]

Supplementary Information

Climate, currents and species traits contribute to early stages of marine species redistribution

Jorge García Molinos, Heather L. Hunt, Madeline E. Green, Curtis Champion, Jason R. Hartog, Gretta T. Pecl

Supplementary Table 1. Variables removed from the initial full models for coastal and latitudinal extension distances during the stepwise procedure based on the fixed effects *p*-values and variation in marginal R2 of the resulting reduced model.

Dependent variable	Model	Variable dropped	*p*	Marginal R2/Conditional R2	Delta Marginal R2
Coastal distance	Full model			0.568/0.839	
	Model 1	Cold spells	0.793	0.573/0.833	-0.035
	Model 2	Range size	0.594	0.570/0.844	-0.003
	Model 3	Habitat preference (categorical, 4 levels)	0.766/0.538/ 0.727	0.570/0.840	-0.0006
	Model 4	Climate velocity x thermal displacement	0.467	0.566/0.847	-0.004
	Model 5	Thermal displacement	0.961	0.562/0.833	-0.003
	Model 6	Mobility (categorical, 2 levels)	0.293	0.568/0.820	0.005
	Model 7	Climate velocity x directional agreement	0.251	0.565/0.816	-0.0025
	Model 8	Life history (categorical, 2 levels)	0.220	0.560/0.820	-0.0044
	Model 9	Trophic category (categorical, 3 levels)	0.132/0.380	0.570/0.796	0.014

Latitudinal distance	Full model			0.510/0.817	
	Model 1	Climate velocity x directional agreement	0.954	0.510/0.817	-0.0001
	Model 2	Climate velocity x thermal displacement)	0.751	0.507/0.820	-0.002
	Model 3	Thermal displacement	0.911	0.513/0.805	0.006
	Model 4	Range size	0.533	0.506/0.822	-0.007
	Model 5	Life history (categorical, 2 levels	0.497	0.502/0.826	-0.003
	Model 6	Cold spells	0.254	0.517/0.813	0.015
	Model 7	Habitat preference (categorical, 4 levels)	0.937/0.186/ 0.585	0.511/0.809	-0.006
	Model 8	Mobility (categorical, 2 levels)	0.156	0.519/0.783	0.007
	Model 9	Position in water column (categorical, 3 levels)	0.526/0.091	0.482/0.756	-0.037

Supplementary Table 2. Model output for final log-linked GLMM models for maximum annual coastal and latitudinal extension distances. Estimates of coefficients are on a log10 scale.

Dependent variable	Variable	Coefficient	SE	DF	*t*	*p*	Marginal R^2 / Conditional R^2
**Coastal distance**	Intercept	5.66	0.26	60	21.34	<0.0001	0.570 / 0.796
	Climate velocity	0.03	0.06	58	0.54	0.589	
	Directional agreement	-1.82	0.42	58	-4.31	0.001	
	Kinetic energy currents	-8.46	2.79	58	-3.03	0.004	
	Position [demersal]	-0.23	0.26	58	-0.92	0.363	
	Position [pelagic]	0.70	0.34	58	2.08	0.042	
	Directional agreement x Kinetic energy	131.36	21.01	58	6.25	<0.0001	
	Directional agreement x Kinetic energy x Climate velocity	-95.41	12.71	58	-7.50	<0.0001	
	**Random effects**						
	σ^2	0.15					

	τ_{00} SPECIES	0.16					
**Latitudinal distance**	Intercept	4.76	0.21	59	21.19	<0.0001	0.482 / 0.756
	Climate velocity	0.12	0.06	59	1.92	0.060	
	Directional agreement	-1.33	0.44	59	-3.03	0.003	
	Kinetic energy	-5.02	2.85	59	-1.76	0.083	
	Trophic category [omnivore]	0.53	0.25	60	2.12	0.039	
	Trophic category [carnivore]	0.41	0.19	59	2.19	0.033	
	Directional agreement x kinetic energy	96.54	21.26	59	4.54	<0.0001	
	Climate velocity x Directional agreement x Kinetic energy	-72.52	12.88	59	-5.63	<0.0001	
	**Random effects**						
	σ^2	0.15					
	τ_{00} SPECIES	0.17					

Supplementary Table 3. Descriptions of oceanographic variables used to derive current strength, velocity of climate change and directional agreement predictors of out-of-range species observations.

Oceanographic variable	Description	Temporal range	Units
SST	Sea surface temperature from Advanced Very High Resolution Radiometer (AVHRR) processed by CSIRO Oceans and Atmosphere as 3-day composite layers with 0.04° spatial resolution	*January 1993 – September 2014*	°C
	Sea surface temperature from the Regional Australian Multi-Sensor Sea surface temperature Analysis processed by the Australian Bureau of Meteorology with 0.08° spatial resolution	*March 2009 – December 2018*	
EKE	Eddy kinetic energy derived from altimetry processed by CSIRO Oceans and Atmosphere as EKE Australasia with 0.2° spatial resolution	*January 1993 – December 2018*	$m^2 s^{-2}$
Current direction	Climatology of directional vectors of current velocity from the United States National Oceanographic and Atmospheric Administration with 0.25 spatial resolution	1979 – December 2018	–

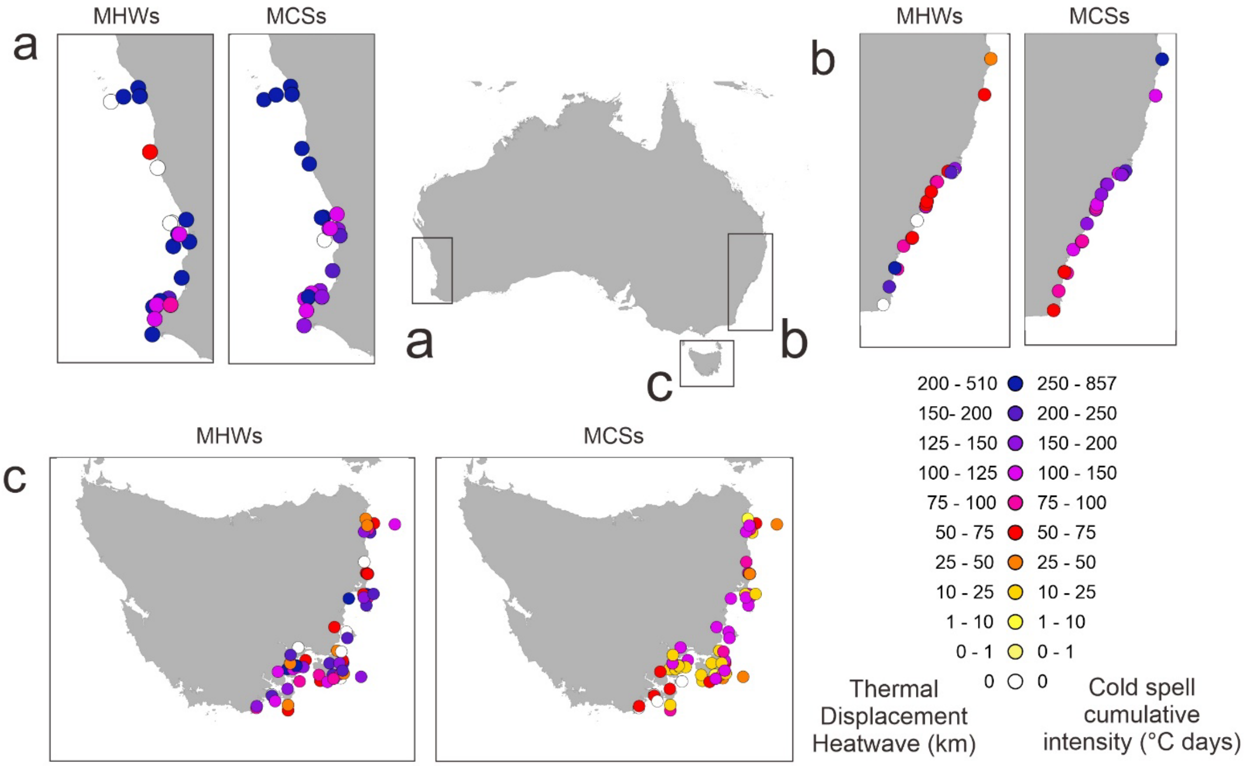

Supplementary Fig. 1. Median thermal displacement associated with marine heatwaves (MHWs) and cumulative intensity of marine cold spells (MCSs) integrated over the 10-year period prior to the year each out-of-range observation was reported in the 3 study regions (see Methods for details on the calculation of both metrics).

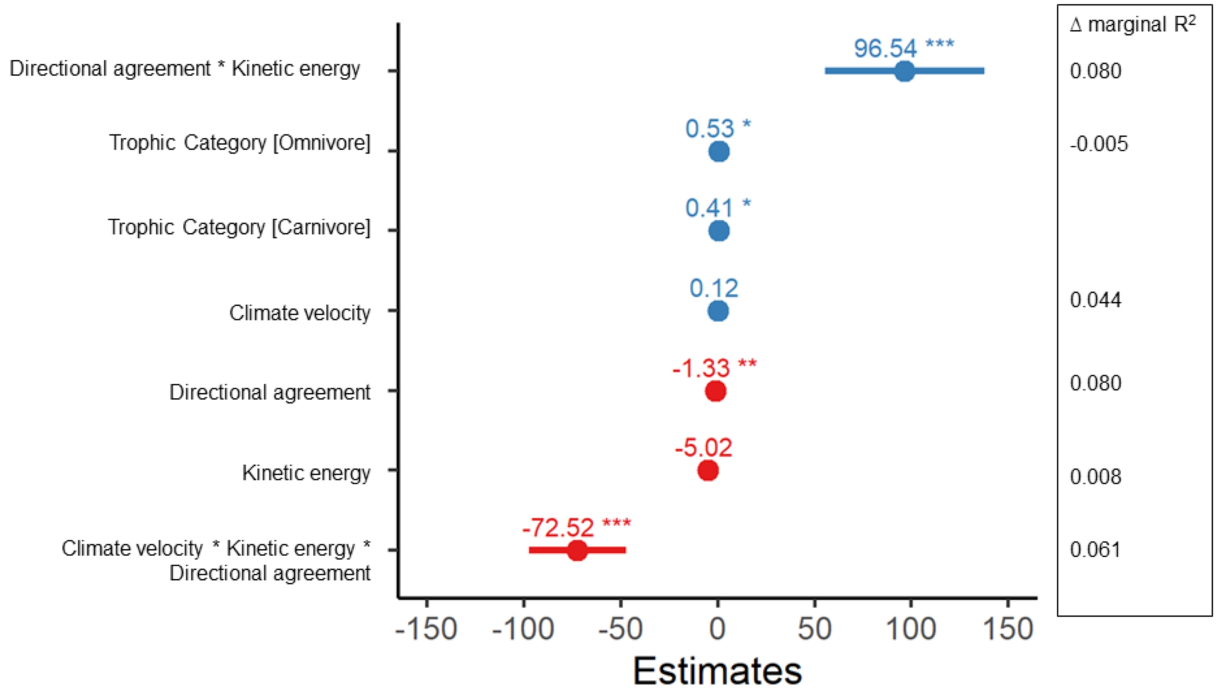

Supplementary Fig. 2. Estimates of regression coefficients with corresponding 95% confidence intervals for each main and interaction term retained in the final log-linked GLMM for prediction of maximum latitudinal annual extension distances. Estimates are on a log10 scale. The column on the right provides the relative change in marginal (fixed effect) R^2 resulting from the removal of that particular term from the final model as an indication of its relative contribution towards the total observed variance in response explained by the selected model.

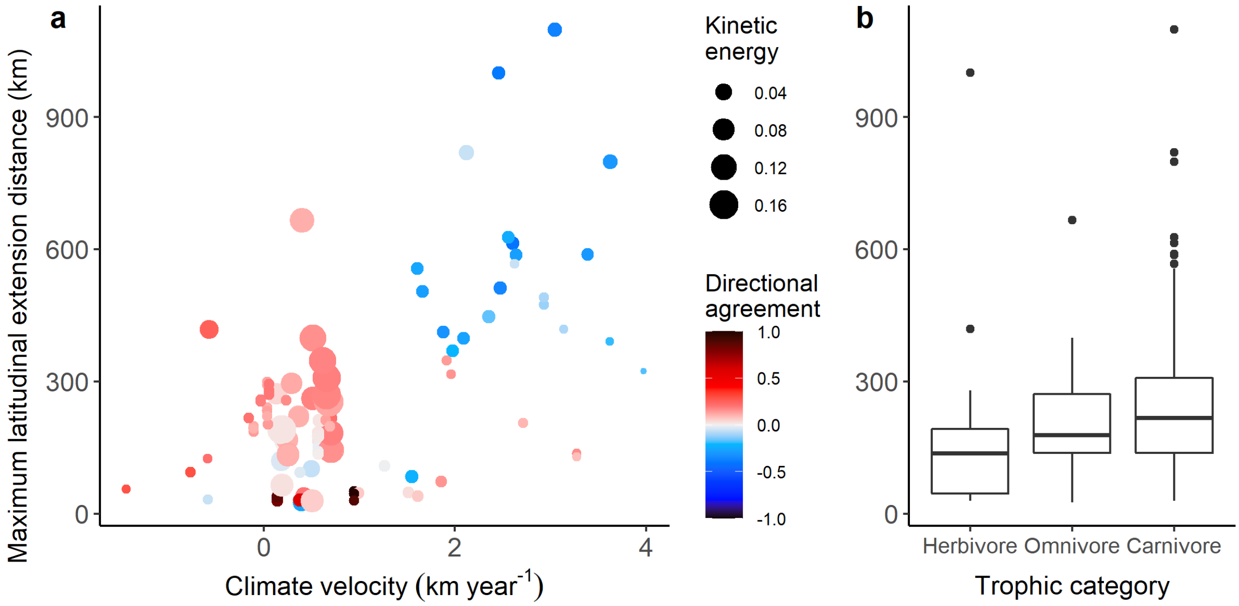

Supplementary Fig. 3. Relationships between observed maximum latitudinal annual extension distances and the predictor variables retained in the final model. (a) Scatterplot of extension distance and climate velocity with colours and size of the points showing the directional agreement between climate velocity, ocean currents and current strength, respectively. (b) Box plots (center line, median; box limits, 75th and 25th percentiles; whiskers, 1.5x interquartile range; points, outliers) of extension distance grouped by trophic level.

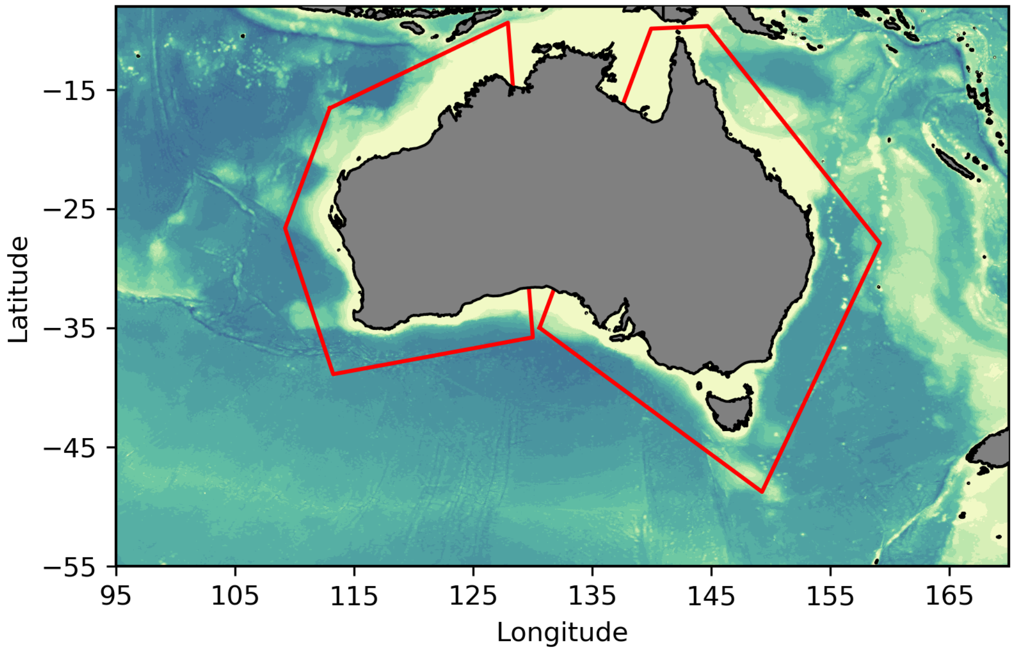

Supplementary Fig. 4. Polygons defining eastern and western Australia for determining historical latitudinal distribution limits of non-target species using occurrences from OBIS and ALA. The background is the GEODATA 9 Second DEM Version 2 digital elevation model (Geoscience Australia and Centre for Resource and Environmental Studies (CRES), the Australian National University).

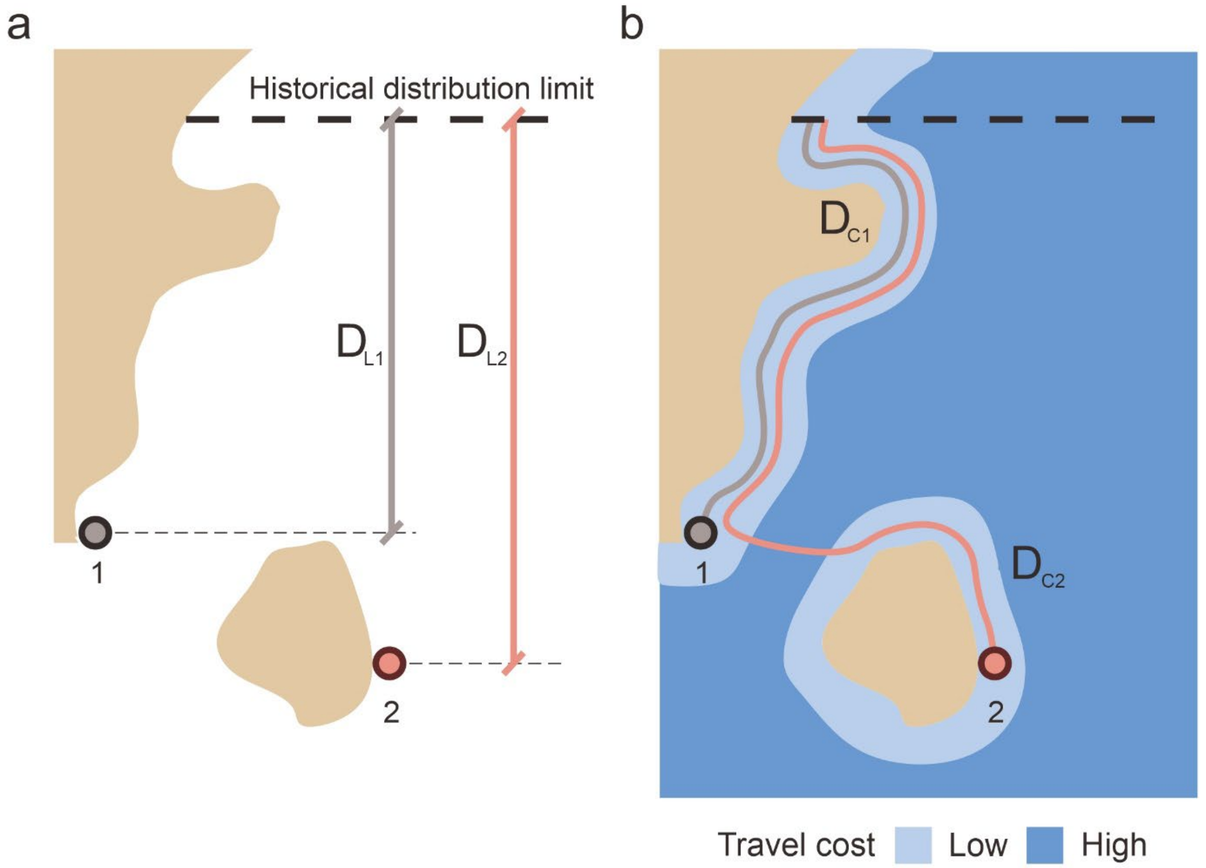

Supplementary Fig. 5. Schematic showing the differences between the two metrics used for estimating maximum annual out-of-range extension distances: (a) latitudinal distances (D_L) calculated as differences in latitude between an observation and the historical southern latitude for the corresponding species, and (b) along-the-coast distances (D_C) calculated as least-cost-path distances constrained to the coastline between the historical southern limit and the observation. Note how the high travel cost associated with open water keeps the least-cost-path to the coast whenever possible yet allows cross-over where needed to reach the destination point.
