# Supplementary material for: Climate, currents and species traits contribute to early stages of marine species redistribution
Source: Commun Biol. 2022 Dec 3;5:1329. doi: 10.1038/s42003-022-04273-0 (PMC9719494; doi:10.1038/s42003-022-04273-0)
Supplement: Supplementary file 2 — Description of Additional Supplementary Files [file 42003_2022_4273_MOESM2_ESM.pdf]

Description of Additional Supplementary Files

File name: Supplementary Data 1

Description: The source data behind the analyses and graphs in the paper. Maximum annual out-of-range observations, calculated extension distances and explanatory variables. An explanation of the column names and descriptions of the corresponding variables are provided in the metadata tab.

File name: Supplementary Data 2

Description: Biological trait data for all species represented in the maximum annual out-of-range observation dataset. Excel data file of species names, historical latitudinal distribution limits and biological traits. References used to determine traits and historical distribution limits are listed.
