# Supplementary material for: Climate, currents and species traits contribute to early stages of marine species redistribution
Source: Commun Biol. 2022 Dec 3;5:1329. doi: 10.1038/s42003-022-04273-0 (PMC9719494; doi:10.1038/s42003-022-04273-0)
Supplement: Supplementary file 5 — Reporting Summary [file 42003_2022_4273_MOESM5_ESM.pdf]

nature portfolio

Corresponding author(s): Heather Hunt

Last updated by author(s): Oct 28, 2022

Reporting Summary

Nature Portfolio wishes to improve the reproducibility of the work that we publish. This form provides structure for consistency and transparency in reporting. For further information on Nature Portfolio policies, see our Editorial Policies and the Editorial Policy Checklist.

Statistics

For all statistical analyses, confirm that the following items are present in the figure legend, table legend, main text, or Methods section.

n/a	Confirmed	
☐	☒	The exact sample size (*n*) for each experimental group/condition, given as a discrete number and unit of measurement
☒	☐	A statement on whether measurements were taken from distinct samples or whether the same sample was measured repeatedly
☐	☒	The statistical test(s) used AND whether they are one- or two-sided<br>*Only common tests should be described solely by name; describe more complex techniques in the Methods section.*
☐	☒	A description of all covariates tested
☐	☒	A description of any assumptions or corrections, such as tests of normality and adjustment for multiple comparisons
☐	☒	A full description of the statistical parameters including central tendency (e.g. means) or other basic estimates (e.g. regression coefficient) AND variation (e.g. standard deviation) or associated estimates of uncertainty (e.g. confidence intervals)
☐	☒	For null hypothesis testing, the test statistic (e.g. *F*, *t*, *r*) with confidence intervals, effect sizes, degrees of freedom and *P* value noted<br>*Give P values as exact values whenever suitable.*
☒	☐	For Bayesian analysis, information on the choice of priors and Markov chain Monte Carlo settings
☒	☐	For hierarchical and complex designs, identification of the appropriate level for tests and full reporting of outcomes
☒	☐	Estimates of effect sizes (e.g. Cohen's *d*, Pearson's *r*), indicating how they were calculated

Our web collection on statistics for biologists contains articles on many of the points above.

Software and code

Policy information about availability of computer code

Data collection	Observations (photographs and associated information including latitude and longitude) were submitted by citizen scientists to the Redmap (Range Extension Database and Mapping project) via a website (www.redmap.org.au) or smartphone application.
Data analysis	Statistical analyses were carried out using R version 4.0.2. All R packages and functions used for analyses are listed in the Methods of the paper. Thermal displacements associated with marine heatwaves were estimated in MATLAB using a modified version of the scripts provided by Jacox et al. (2020). The modified code is available on request.

For manuscripts utilizing custom algorithms or software that are central to the research but not yet described in published literature, software must be made available to editors and reviewers. We strongly encourage code deposition in a community repository (e.g. GitHub). See the Nature Portfolio guidelines for submitting code & software for further information.

Data

Policy information about availability of data

All manuscripts must include a data availability statement. This statement should provide the following information, where applicable:

- Accession codes, unique identifiers, or web links for publicly available datasets
- A description of any restrictions on data availability
- For clinical datasets or third party data, please ensure that the statement adheres to our policy

All source data for the analyses are included in the Supplementary Data files for this article. All other data are available from the corresponding author on reasonable request.

Field-specific reporting

Please select the one below that is the best fit for your research. If you are not sure, read the appropriate sections before making your selection.

☐ Life sciences ☐ Behavioural & social sciences ☒ Ecological, evolutionary & environmental sciences

For a reference copy of the document with all sections, see nature.com/documents/nr-reporting-summary-flat.pdf

Ecological, evolutionary & environmental sciences study design

All studies must disclose on these points even when the disclosure is negative.

Study description	We use scientist-verified out-of-range observations (annual maximum out-of-range distances) from a national citizen-science initiative to assess the combined effect of long-term warming (velocity of climate change), climate extremes (i.e., thermal displacements associated with marine heatwaves and cumulative intensity of local marine cold spells), strength (kinetic energy) of ocean currents and their directional agreement with climate velocity, and species traits on early stages of marine range extensions in two warming 'hotspot' regions of southern Australia.
Research sample	Annual maximum out-of-range distances calculated both as latitudinal distance (i.e., direct latitudinal extension distance) and distance along the coastline (i.e., coastal extension distance) relative to the poleward historical range boundaries of 61species of marine fishes, reptiles, and invertebrates in Australia
Sampling strategy	Data are citizen science observations submitted to the Range Extension Database and Mapping citizen science program Redmap Australia www.redmap.org.au.
Data collection	Out-of-range observations of marine species in Australia were obtained from Redmap (Range Extension Database and Mapping project, www.redmap.org.au). Redmap is a citizen science project that requests submission of photographs and associated data for 'unusual' observations of marine species (i.e. out-of-range observations) in Australia to a website or smartphone application. Photographs of each observation are verified by one of a panel of 80 expert scientists.
Timing and spatial scale	Our dataset encompasses observations recorded along the south-eastern and south-western coasts of Australia between 2009 (start of Redmap program) and 2018 (start of data analysis). Observations from northern Australia (i.e., north of 28°S) were excluded due to the insufficient number of citizen science observations in northern regions.
Data exclusions	Only sightings > 20 km from the maximum southern latitude were considered as range extensions in order be conservative due to the possibility of lack of detection of rare individuals when determining the maximum southern latitude. We excluded multiple extension observations per year of a given species as they could be re-sightings of the same individual or result from the same settlement event. The maximum extension distance of each species in a given year formed our response variable in the analyses. Nine of 136 maximum annual out-or-range observations were excluded from analysis as they had a predominantly longitudinal component (i.e., longitudinal out-of-range extension >> latitudinal extension; n=7) or because the path followed between mainland Australia and Tasmania was unclear (n=2), making determination of a path routing uncertain (final n=127 maximum annual extension distances).
Reproducibility	Data analyzed were field observations and not an experiment that could be repeated
Randomization	Randomization was not relevant to our study as organisms were not allocated to groups (observational study)
Blinding	Blinding was not relevant to our study as organisms were not allocated to treatments (observational study)

Did the study involve field work? ☐ Yes ☒ No

Reporting for specific materials, systems and methods

We require information from authors about some types of materials, experimental systems and methods used in many studies. Here, indicate whether each material, system or method listed is relevant to your study. If you are not sure if a list item applies to your research, read the appropriate section before selecting a response.

Materials & experimental systems

n/a	Involved in the study	
☒	☐	Antibodies
☒	☐	Eukaryotic cell lines
☒	☐	Palaeontology and archaeology
☒	☐	Animals and other organisms
☒	☐	Human research participants
☒	☐	Clinical data
☒	☐	Dual use research of concern

Methods

n/a	Involved in the study	
☒	☐	ChIP-seq
☒	☐	Flow cytometry
☒	☐	MRI-based neuroimaging
